# Supplementary material for: Novel Complexes of 3-[3-(1H-Imidazol-1-yl)propyl]-3,7-diaza-bispidines and β-Cyclodextrin as Coatings to Protect and Stimulate Sprouting Wheat Seeds
Source: Molecules. 2022 Nov 1;27(21):7406. doi: 10.3390/molecules27217406 (PMC9655490; doi:10.3390/molecules27217406)
Supplement: Supplementary file 1 [file molecules-27-07406-s001.zip › molecules-1973704-supplementary.pdf]

Article

Novel Complexes of 3-[3-(1*H*-Imidazol-1-yl)propyl]-3,7-diazabispidines and β-Cyclodextrin as Coatings to Protect and Stimulate Sprouting Wheat Seeds

Altynay B. Kaldybayeva[1,2], Valentina K. Yu[1,*], Aigul E. Malmakova[1], Tamara Li[3], Assel Yu. Ten[1], Tulegen M. Seilkhanov[4], Kaldybay D. Praliyev[1], K. Darrell Berlin[5]

[1] Laboratory of Chemistry of Synthetic and Natural Medicinal Substances, A.B. Bekturov Institute of Chemical Sciences,106 Sh.Ualikhanov St., Almaty, 050010, Kazakhstan

[2] Department of Chemistry and Technology of Organic Substances, Natural Compounds and Polymers, Al Farabi Kazakh National University, 71 al-Farabi Ave, Almaty, 050040, Kazakhstan

[3] Laboratory of Cell Engineering, Institute of Plant Biology and Biotechnology, 45,Timiryazev str., Almaty, 050040, Kazakhstan

[4] Laboratory of the Engineering Profile of NMR Spectroscopy, Sh.UalikhanovKokshetau University, 76, Abay St., Kokshetau, 020000, Kazakhstan

[5] Department of Chemistry, Oklahoma State University, Stillwater, OK 74078, USA

* Correspondence: yu_vk@mail.ru (V.K.Y.)

Supplementary Material

Spectral IR data section ……………………………..….Figures S1–S6

Spectral NMR data section…………………………….Figures S7–S18

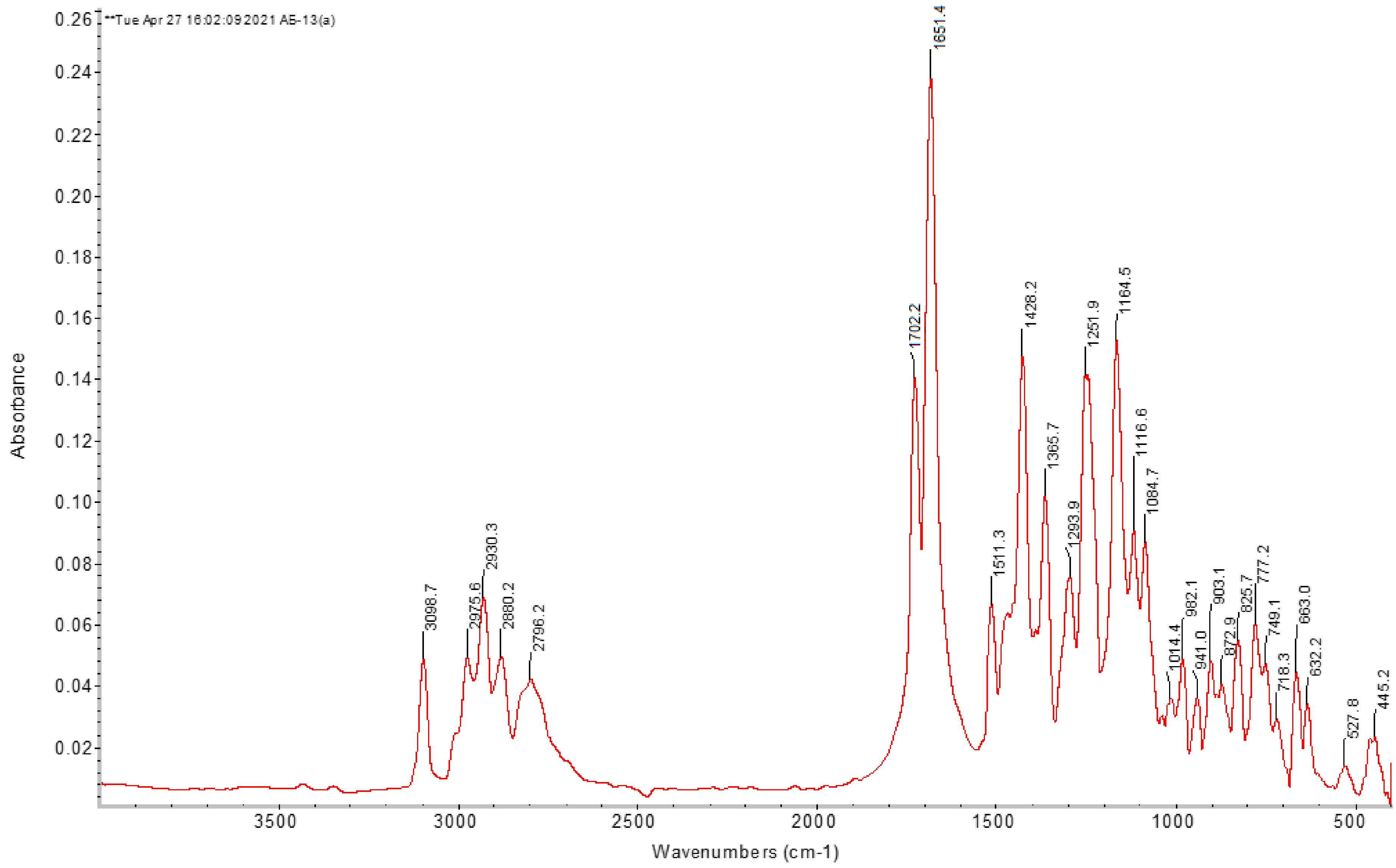

Figure S1. IR spectrum of 3-Boc-7-[3-(1*H*-imidazol-1-)propyl]-3,7-diazabicyclo[3.3.1]nonan-9-one (6)

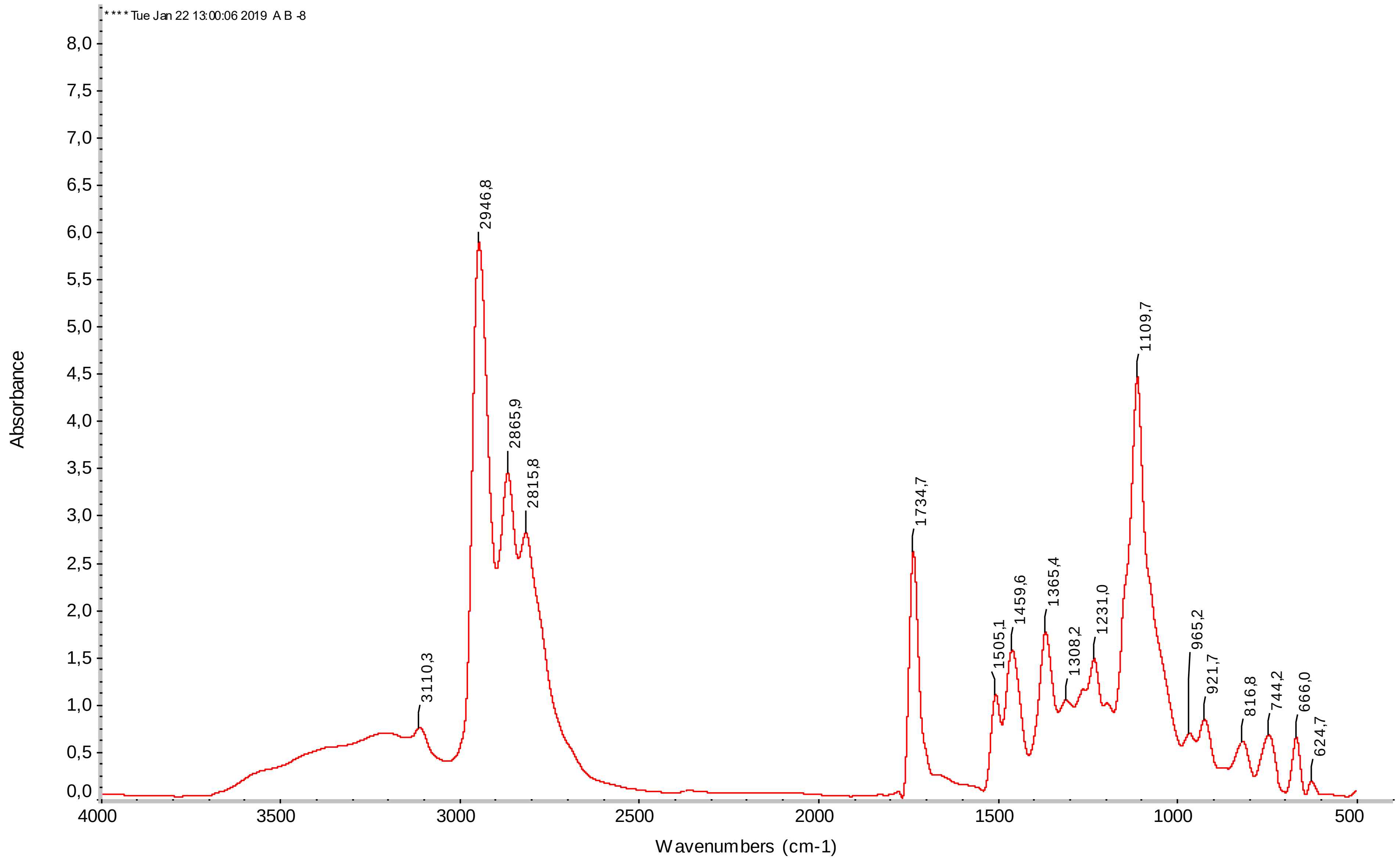

Figure S2. IR spectrum of 3-(3-butoxypropyl)-7-[3-(1*H*-imidazol-1-yl)propyl]-3,7-diazabicyclo[3.3.1]nonan-9-one **(7)**

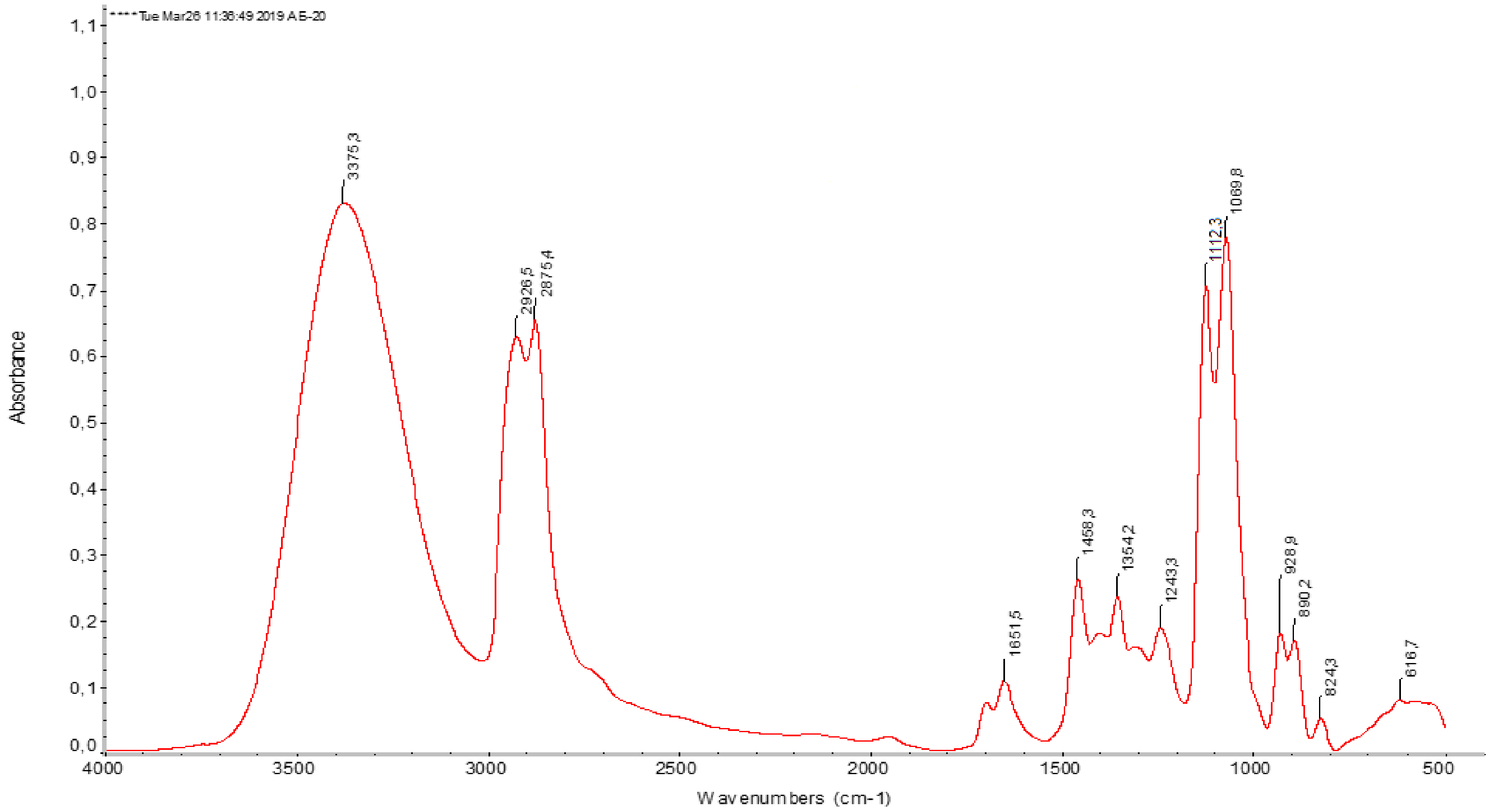

Figure S3. IR spectrum of 3-Boc-7-[3-(1*H*-imidazol-1-yl)propyl]-3,7-diazabicyclo[3.3.1]nonane (8)

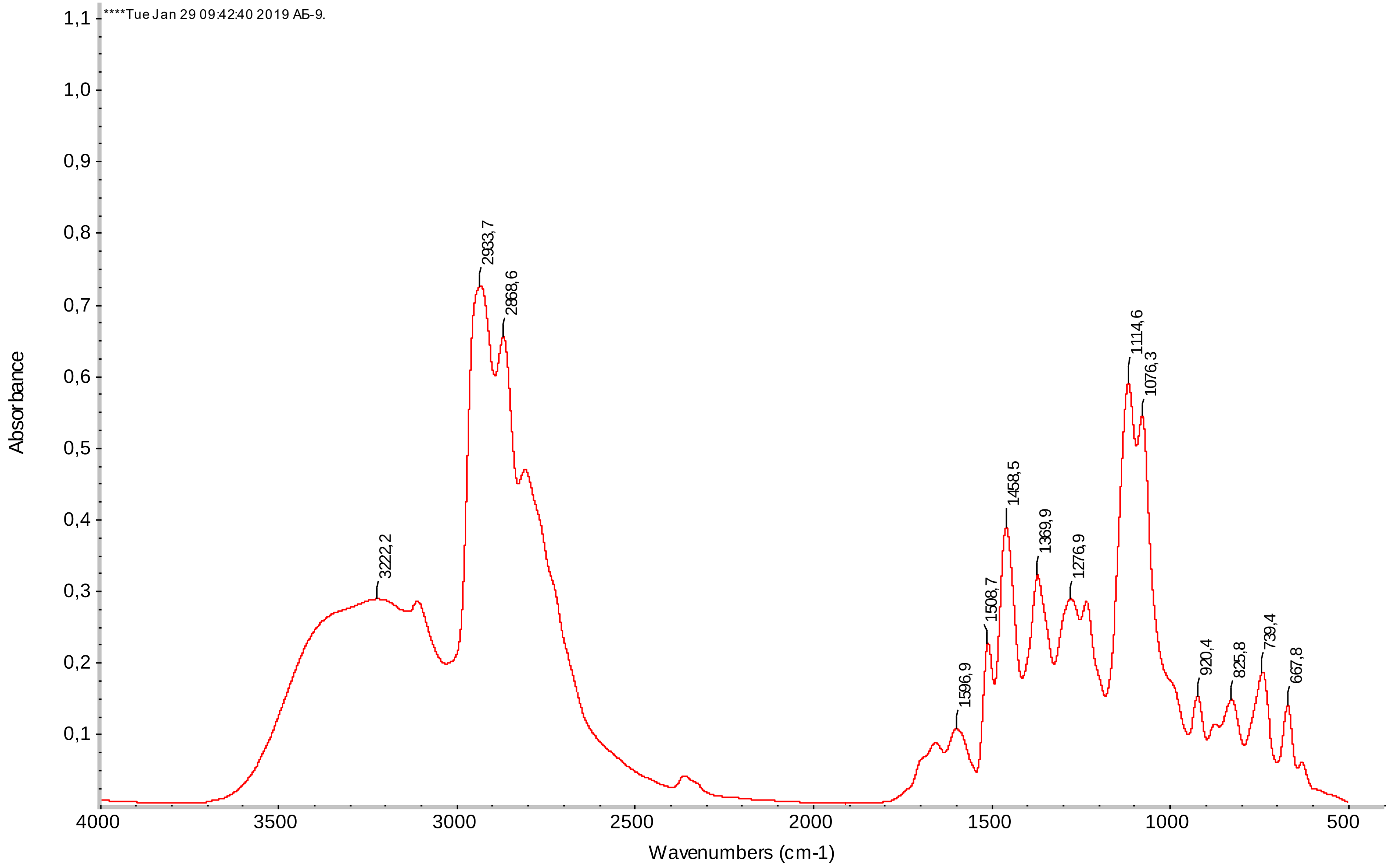

Figure S4. IR spectrum of 3-(3-butoxypropyl)-7-[3-(1*H*-imidazol-1-yl)propyl]-3,7-diazabicyclo[3.3.1]nonane (10)

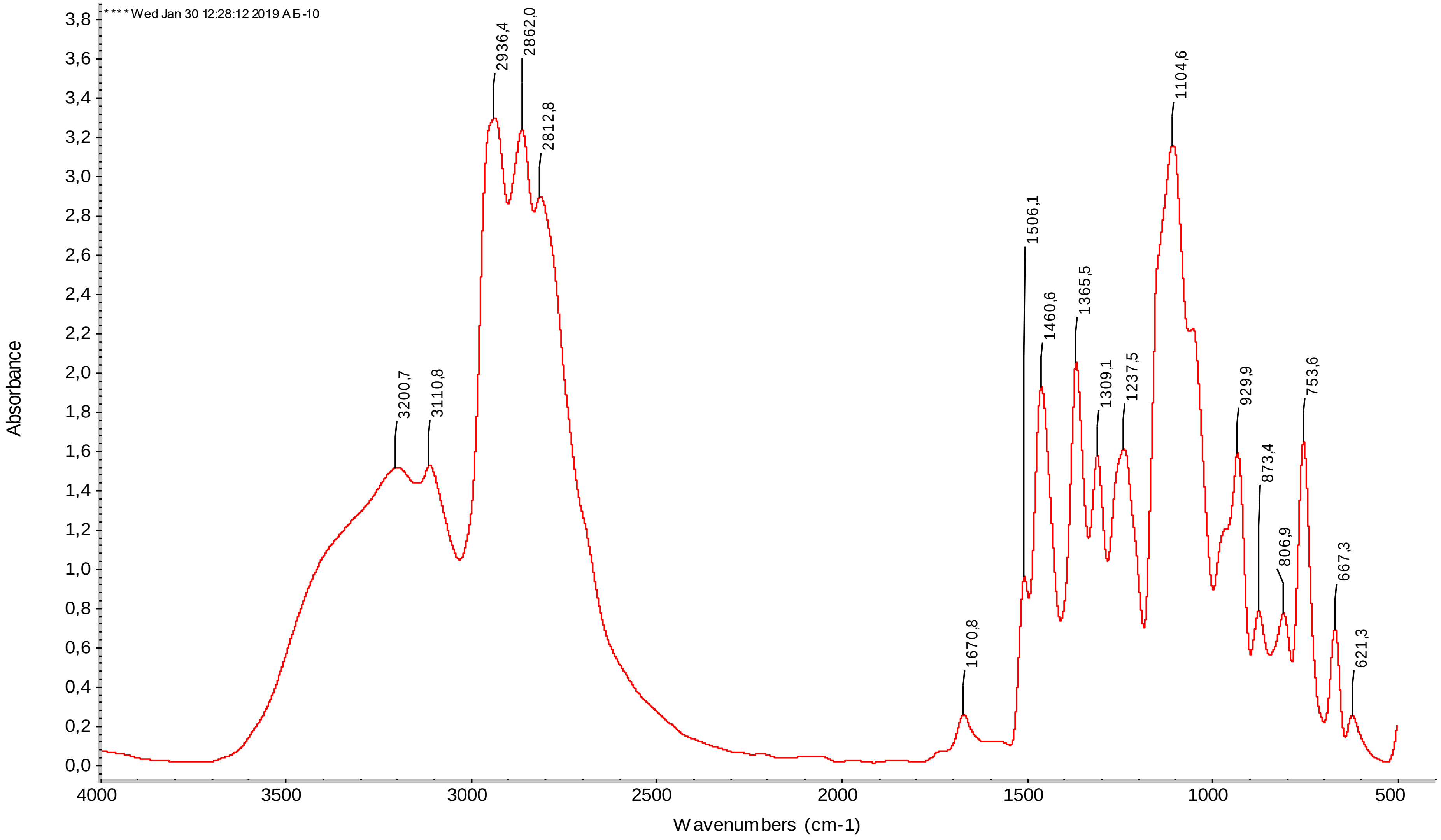

Figure S5. IR spectrum of oxime of 3-(3-butoxypropyl)-7-[3-(1*H*-imidazol-1-ylpropyl-3,7-diazabicyclo[3.3.1]nonane-9-one (12)

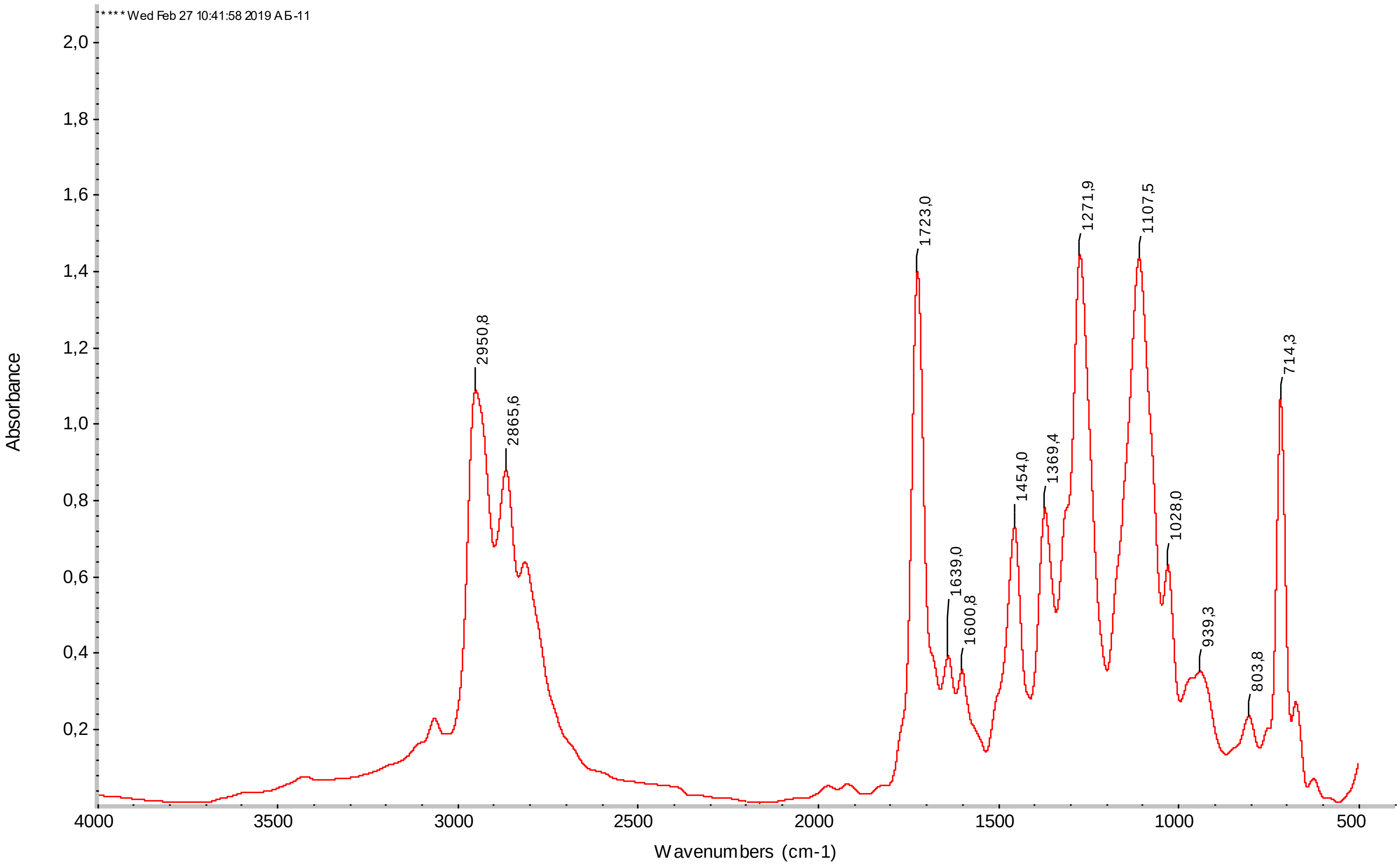

Figure S6. IR spectrum of O-benzoyloxime of 3-(3-butoxypropyl)-7-[3-(1*H*-imidazol-1yl)propyl-3,7-diazabicyclo[3.3.1]nonan-9-one (13)

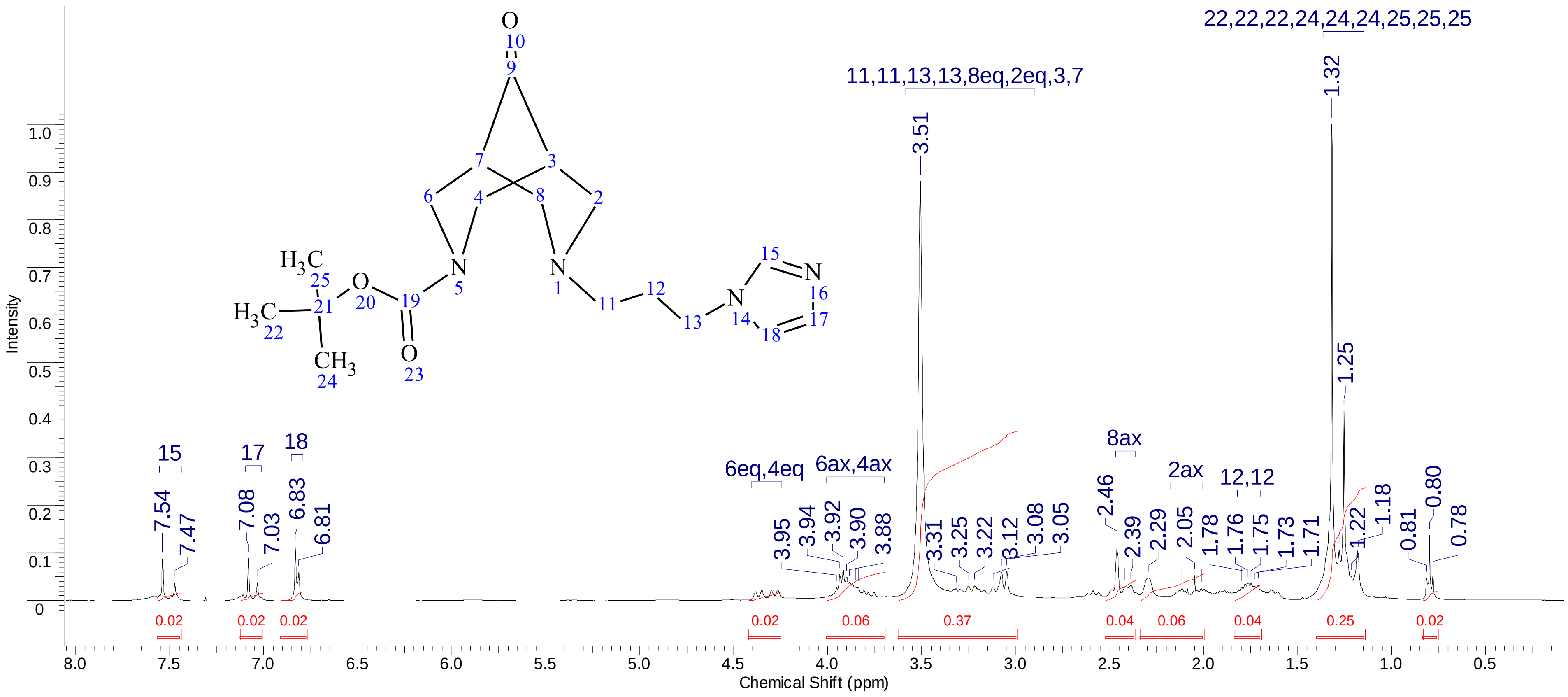

No.	Annotation	(ppm)	(Hz)	Height	No.	Annotation	(ppm)	(Hz)	Height
1	22,22,22,24,24,24,25,25,25	1.22-1.32	527.1	1.0000	7	18	6.81-6.83	2731.2	0.1122
2	12	1.71-1.78	711.6	0.0348	8	17	7.03-7.08	2831.0	0.0880
3	2ax, 8ax	2.05-2.46	983.6	0.1196	9	15	7.47-7.54	3013.7	0.0870
4	11,11,13,13,2eq,8eq,3,7	3.05-3.51	1402.2	0.8804					
5	4ax,6ax	3.88-3.95	1580.3	0.0264					
6	4eq,6eq	4.24-4.41	2120.2	0.0545					

Figure S7. ^{1}H NMR spectrum of 3-Boc-7-[3-(1*H*-imidazol-1-yl)propyl]-3,7-diazabicyclo[3.3.1]nonan-9-one (6) in DMSO-d$_6$

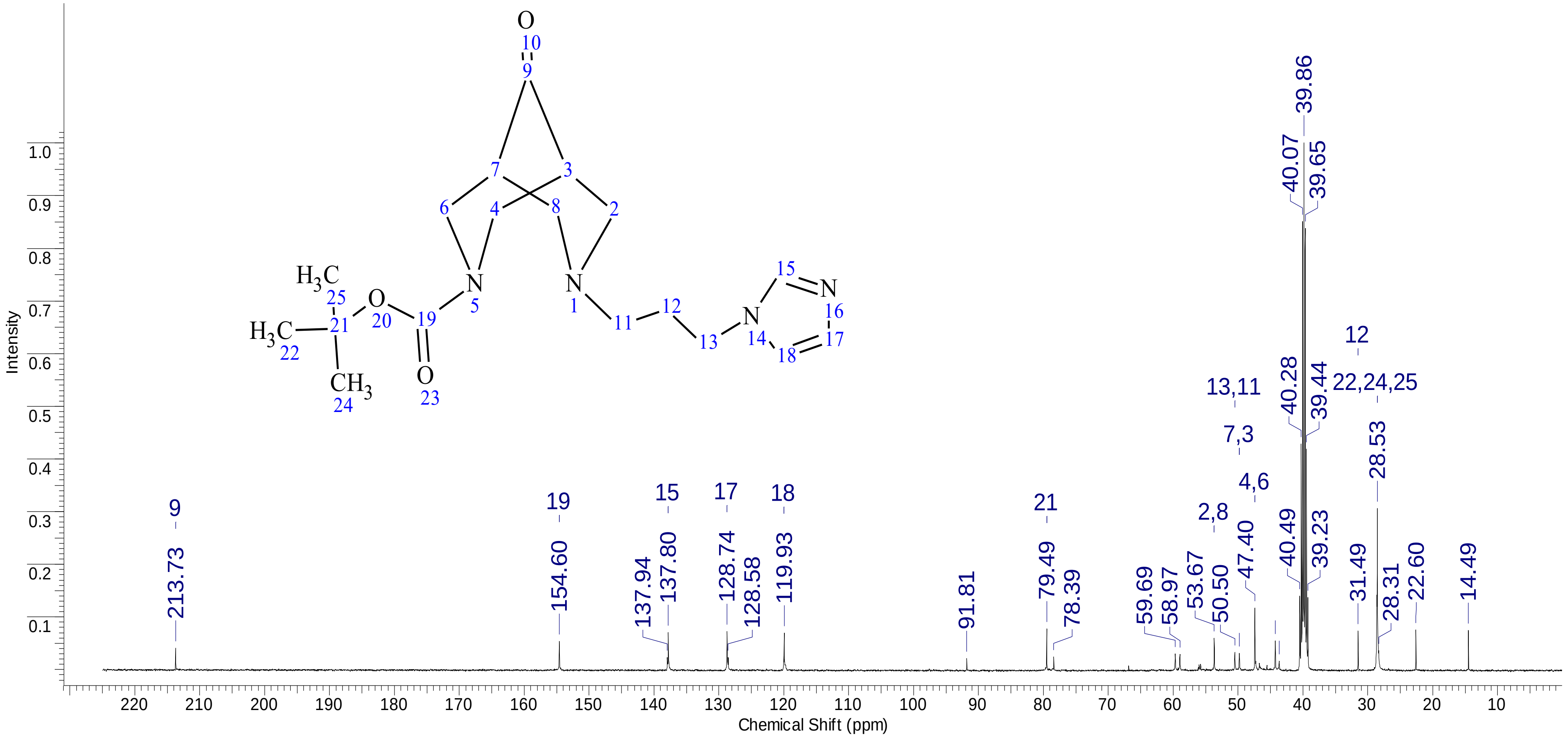

No.	Annotation	(ppm)	(Hz)	Height	No.	Annotation	(ppm)	(Hz)	Height
1	22,24,25	28.5	2867.9	0.3060	7	21	79.5	7990.9	0.0776
2	12	31.5	3165.9	0.0739	8	18	119.9	12065.3	0.0241
3	4,6	47.4	4764.7	0.1170	9	17	128.7	12941.3	0.0725
4	3,7	49.8	5007.2	0.0306	10	15	137.8	13851.9	0.0707
5	11,13	50.5	5076.2	0.0331	11	19	154.6	15541.7	0.0540
6	2,8	53.7	5395.3	0.0600	12	9	213.7	21485.1	0.0412

Figure S8. ^{13}C NMR spectrum of 3-Boc-7-[3-(1*H*-imidazol-1-yl)propyl]-3,7-diazabicyclo[3.3.1]nonan-9-one (6) in DMSO-d_6

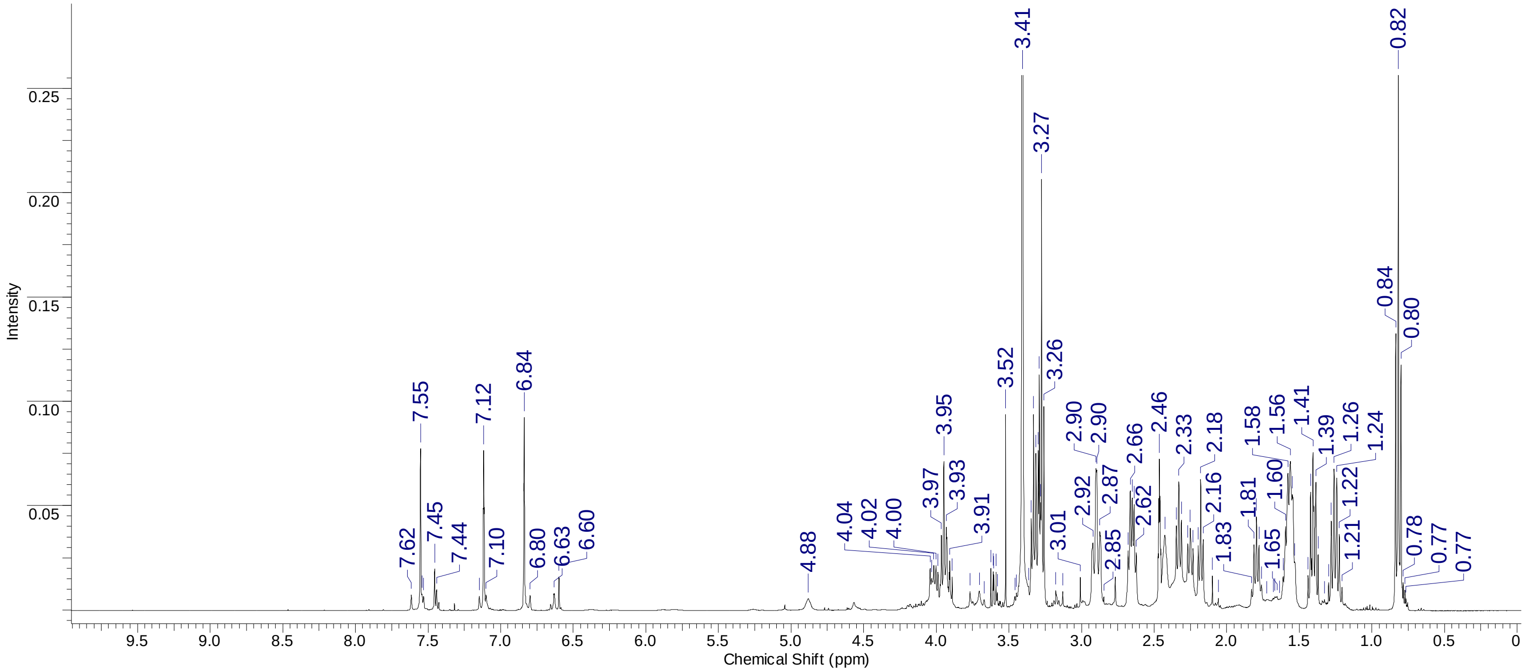

No.	Annotation	(ppm)	(Hz)	Height	No.	Annotation	(ppm)	(Hz)	Height
1	26	0.84	334.3	0.1325	10	2eq, 8eq	3.01	1202.5	0.0157
2	24, 25	1.41	562.3	0.0754	11	11	3.27	1309.2	0.2063
3	12	1.56	624.2	0.0712	12	21	3.41	1361.9	1.0000
4	20	1.65	661.2	0.0066	13	23	3.30	1318.4	0.0764
5	4ax, 6ax	2.18	871.4	0.0626	14	13	3.89-4.04	1561.6	0.0236
6	2ax, 8ax	2.33	931.4	0.0615	15	18	6.80-6.84	2733.9	0.0924
7	19	2.66	1065.2	0.0572	16	17	7.10	2838.3	0.0072
8	3, 7	2.90	1157.7	0.0670	17	15	7.44-7.45	2845.2	0.0764
9	4eq, 6eq	2.90	1159.5	0.0679					

Figure S9. ^{1}H NMR spectrum of 3-(3-butoxypropyl)-7-[3-(1*H*-imidazol-1-yl)propyl]-3,7-diazabicyclo[3.3.1]nonan-9-one (7) in DMSO-d_6

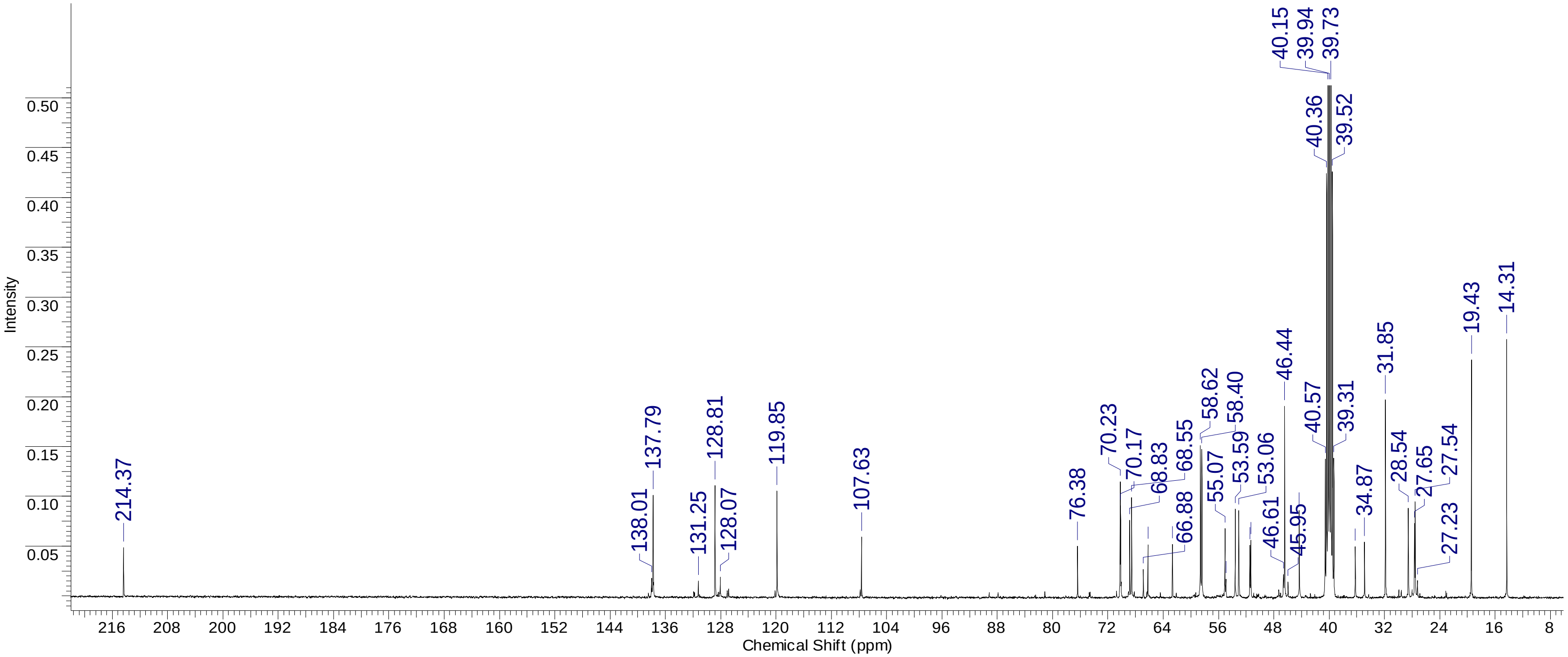

No.	Annotation	(ppm)	(Hz)	Height	No.	Annotation	(ppm)	(Hz)	Height
1	26	14.3	1438.8	0.2575	11	4, 6	66.9	6722.8	0.0265
2	25	19.4	1953.5	0.2369	12	2, 8	68.6	6891.5	0.0987
3	20	27.7	2779.7	0.0728	13	23	70.2	5415.3	0.0124
4	12	28.5	2868.8	0.0881	14	18	119.9	12048.1	0.1054
5	24	31.9	3201.4	0.1970	15	17	131.3	13194.4	0.0150
6	13	46.6	4685.1	0.0217	16	15	137.8	13841.3	0.0136
7	11	53.1	5334.0	0.0859	17	9	214.4	21549.3	0.0486
8	19	53.6	5386.7	0.0871					
9	3, 7	58.4	5870.7	0.1470					
10	21	63.0	4515.2	0.1587					

Figure S10. ^{13}C NMR spectrum of 3-(3-butoxypropyl)-7-[3-(1*H*-imidazol-1-yl)propyl]-3,7-diazabicyclo[3.3.1]nonan-9-one (7) in DMSO-d_6

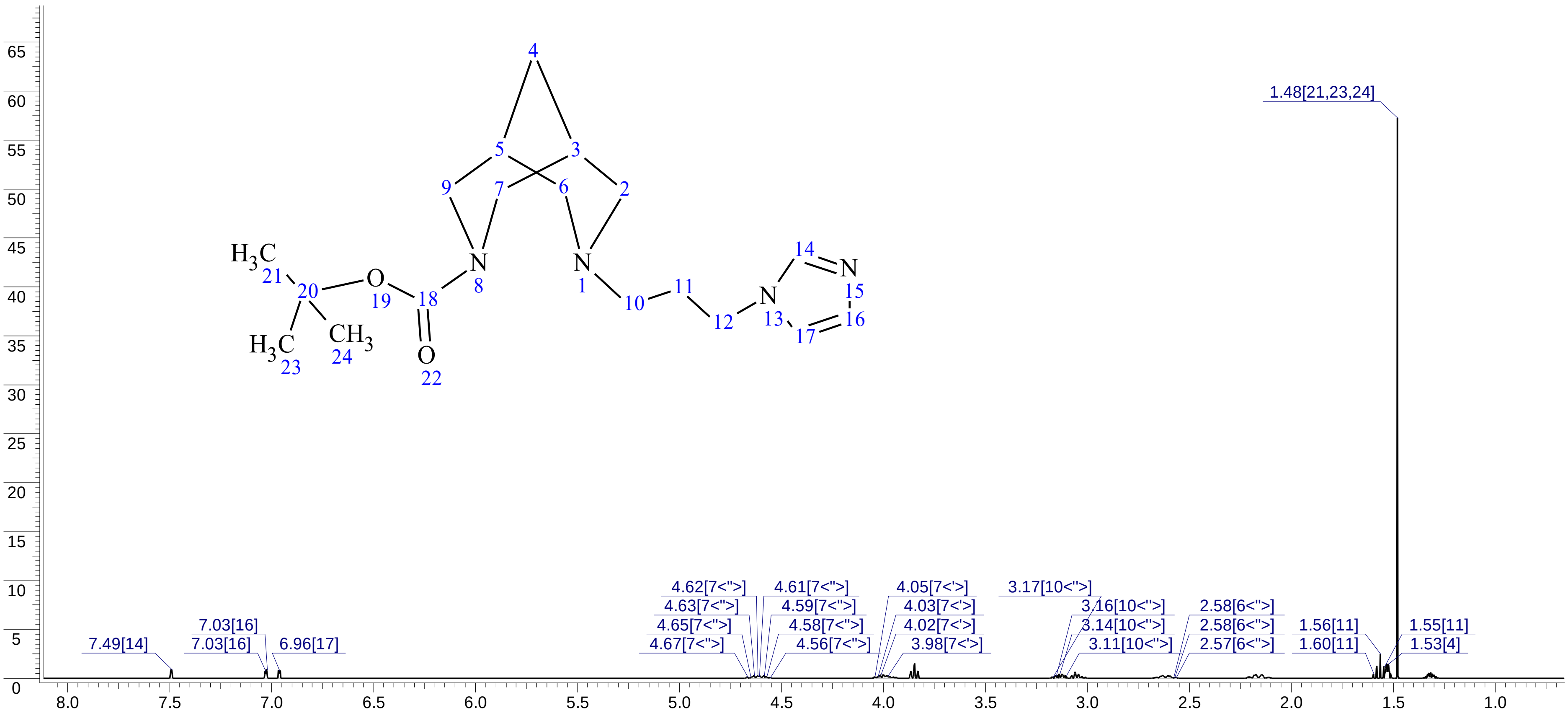

No.	Annotation	(ppm)	No.	Annotation	(ppm)
1	3, 5	1.32	9	12	3.85
2	21, 23, 24	1.48	10	7ax, 9ax	3.98-4.05
3	4	1.53	11	7eq, 9eq	4.56-4.67
4	11	1.55-1.60	12	17	6.96
5	2ax, 6ax	2.16	13	16	7.03
6	2eq, 6eq	2.57-2.58	14	14	7.49
7	10ax	3.05			
8	10eq	3.11-3.17			

Figure S11. ^{1}H NMR spectrum of 3-Boc-7-[3-(1*H*-imidazol-1-yl)propyl]-3,7-diazabicyclo[3.3.1]nonane (8) in DMSO-d_6

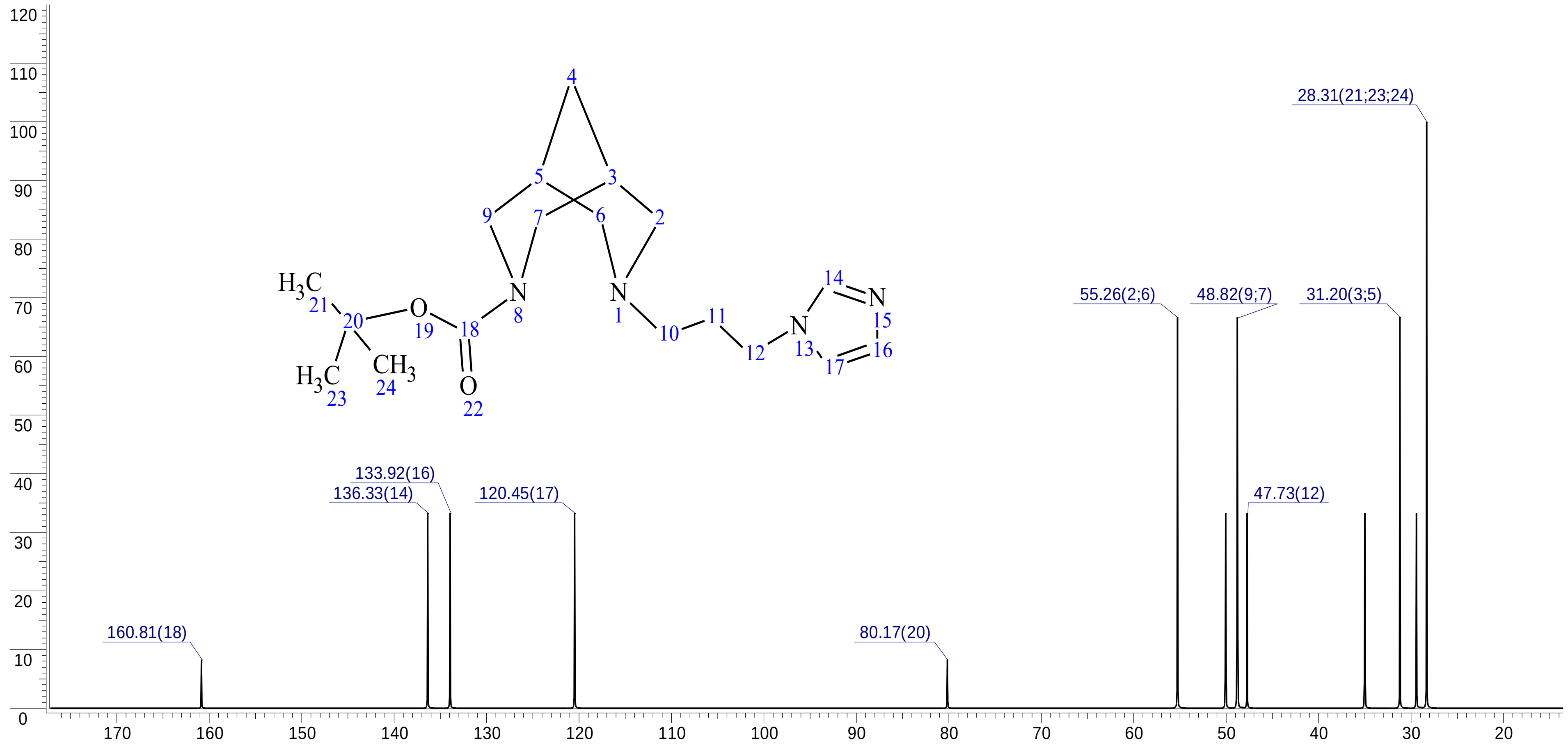

No.	Annotation	(ppm)	No.	Annotation	(ppm)
1	21, 23, 24	28.3	8	2, 6	55.3
2	11	29.4	9	20	80.2
3	3, 5	31.2	10	17	120.5
4	4	35.0	11	16	133.9
5	12	47.7	12	14	136.3
6	7, 9	48.8	13	18	160.8
7	10	50.1			

Figure S12. ^{13}C NMR spectrum of 3-Boc-7-[3-(1*H*-imidazol-1-yl)propyl]-3,7-diazabicyclo[3.3.1]nonane (8) in DMSO-d_6

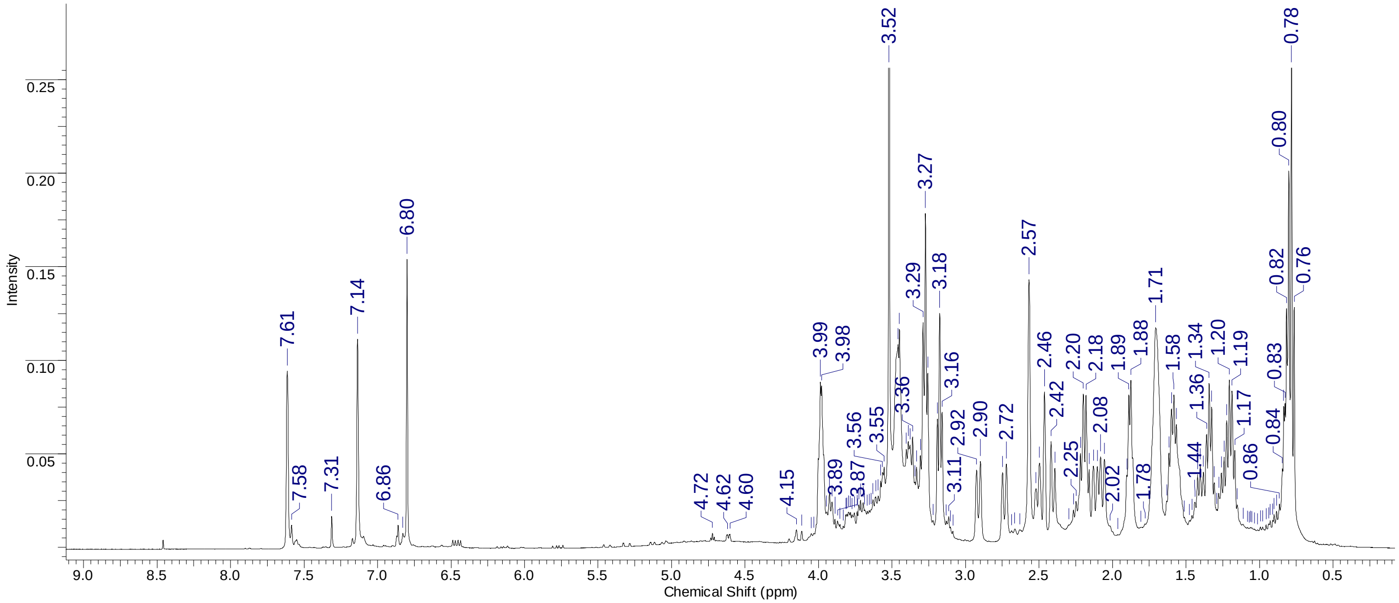

No.	Annotation	(ppm)	(Hz)	Height	No.	Annotation	(ppm)	(Hz)	Height
1	25	0.86	345.7	0.0231	11	2eq, 8eq	2.72	1088.1	0.0447
2	23, 24	1.44	575.2	0.0242	12	10	3.11-3.16	1263.0	0.0722
3	11	1.58	632.4	0.0815	13	20	3.36	1343.1	0.0591
4	19	1.71	682.3	0.1174	14	22	3.38	1350.5	0.0547
5	9	1.88	755.1	0.0815	15	12	3.87	1407.5	0.0245
6	3,7	1.89	457.6	0.0148	16	17	6.86	1678.4	0.0547
7	4ax, 6ax	2.18	871.4	0.0813	17	16	7.14	1789.0	0.0984
8	2ax, 8ax	2.25	898.5	0.0246	18	14	7.61	1876.1	0.0284
9	18	2.46	984.6	0.0832					
10	4eq, 6eq	2.57	1026.7	0.1430					

Figure S13. ^{1}H NMR spectrum of 3-(3-butoxypropyl)-7-[3-(1*H*-imidazol-1-yl)propyl]-3,7-diazabicyclo[3.3.1]nonane (10) in DMSO-d_6

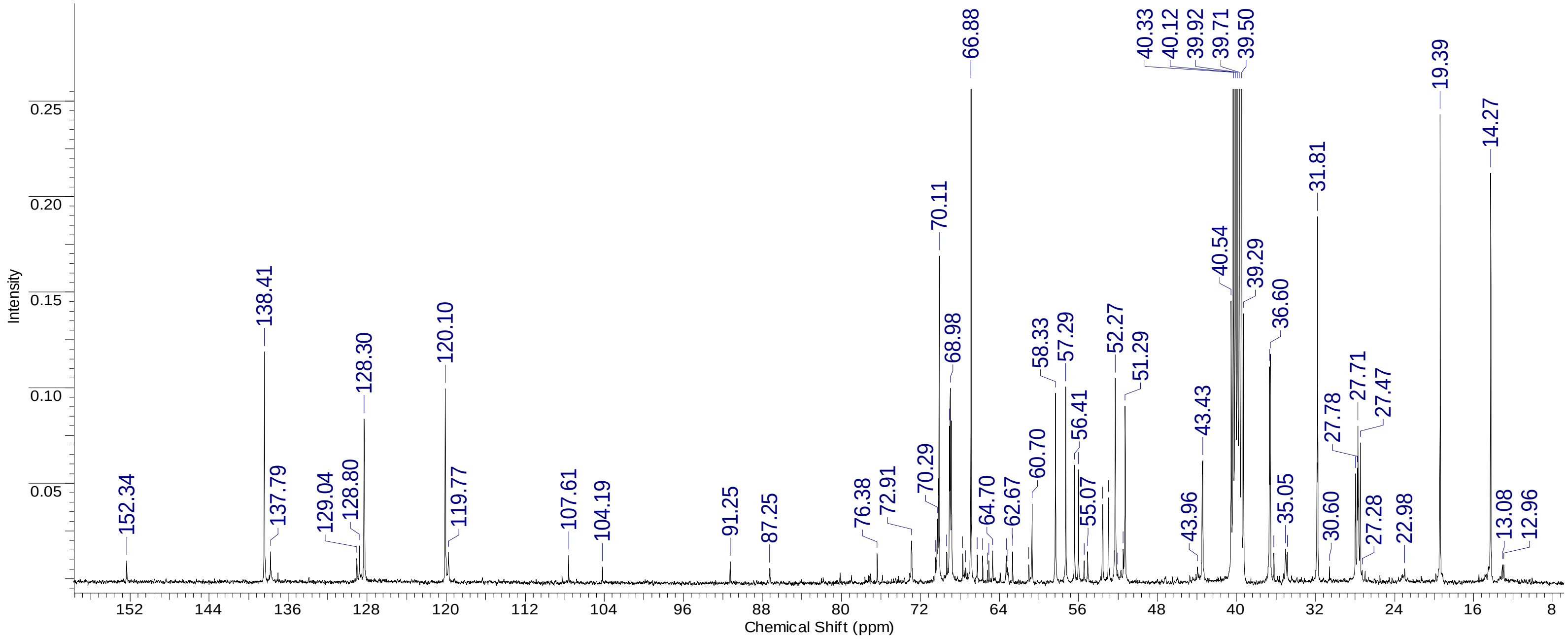

No.	Annotation	(ppm)	(Hz)	Height	No.	Annotation	(ppm)	(Hz)	Height
1	25	14.3	1434.0	0.2122	10	18	52.3	5254.4	0.1048
2	24	19.4	1948.7	0.2430	11	4, 6	60.7	6101.7	0.0391
3	3, 7	27.7	2785.4	0.0798	12	2, 8	62.7	6300.1	0.0140
4	19	27.8	2792.1	0.0579	13	20	66.9	5641.5	0.0145
5	11	30.6	3075.9	0.0064	14	22	70.1	8478.2	0.0154
6	23	31.8	3197.6	0.1893	15	17	120.1	12073.0	0.0994
7	9	35.1	3504.3	0.0136	16	16	129.0	12972.0	0.0107
8	12	44.0	4418.7	0.0061	17	14	137.8	13850.9	0.0141
9	10	51.3	5155.7	0.0903					

Figure S14. ^{13}C NMR spectrum of 3-(3-butoxypropyl)-7-[3-(1*H*-imidazol-1-yl)propyl]-3,7-diazabicyclo[3.3.1]nonane (10) in DMSO-d_6

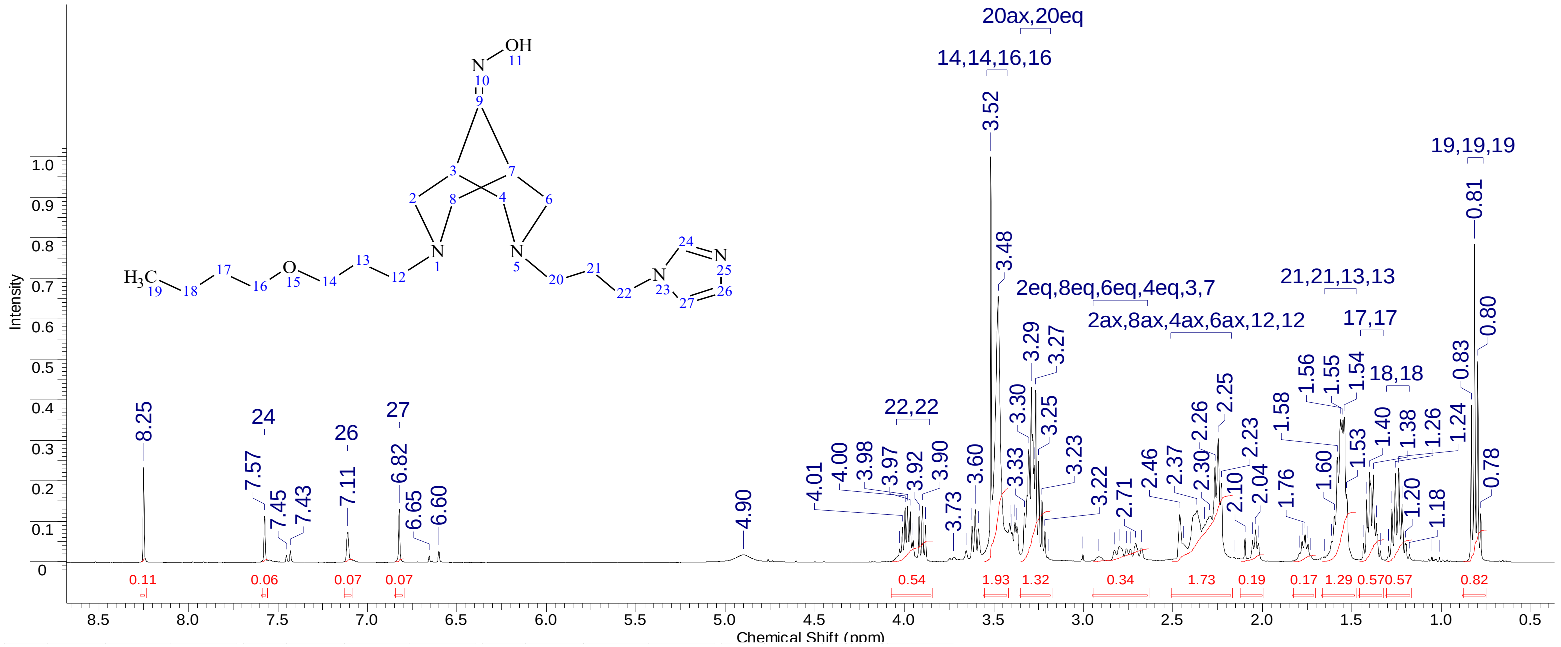

No.	Annotation	(ppm)	(Hz)	Height	No.	Annotation	(ppm)	(Hz)	Height
1	19	0.78-0.83	332.9	0.3869	7	20ax, 20eq	3.23-3.30	1321.1	0.2779
2	18	1.24-1.26	502.3	0.2178	8	14, 16	3.48-3.52	1405.9	1.0000
3	17	1.38-1.40	551.8	0.2147	9	22	3.90-4.01	1603.7	0.0850
4	13, 21	1.53-1.60	638.8	0.1130	10	11, 27	6.82	2727.0	0.1302
5	2ax,4ax,6ax,8ax,12	2.23-2.46	984.1	0.1179	11	26	7.11	2842.4	0.0736
6	2eq,4eq,6eq,8eq,3,7	2.71-2.95	1164.5	0.0128	12	24	7.57	3027.9	0.1135

Figure S15. ^{1}H NMR spectrum of oxime of 3-(3-butoxypropyl)-7-[3-(1*H*-imidazol-1-yl)-propyl]-3,7-diazabicyclo[3.3.1]nonane-9-one (12) in DMSO-d_6

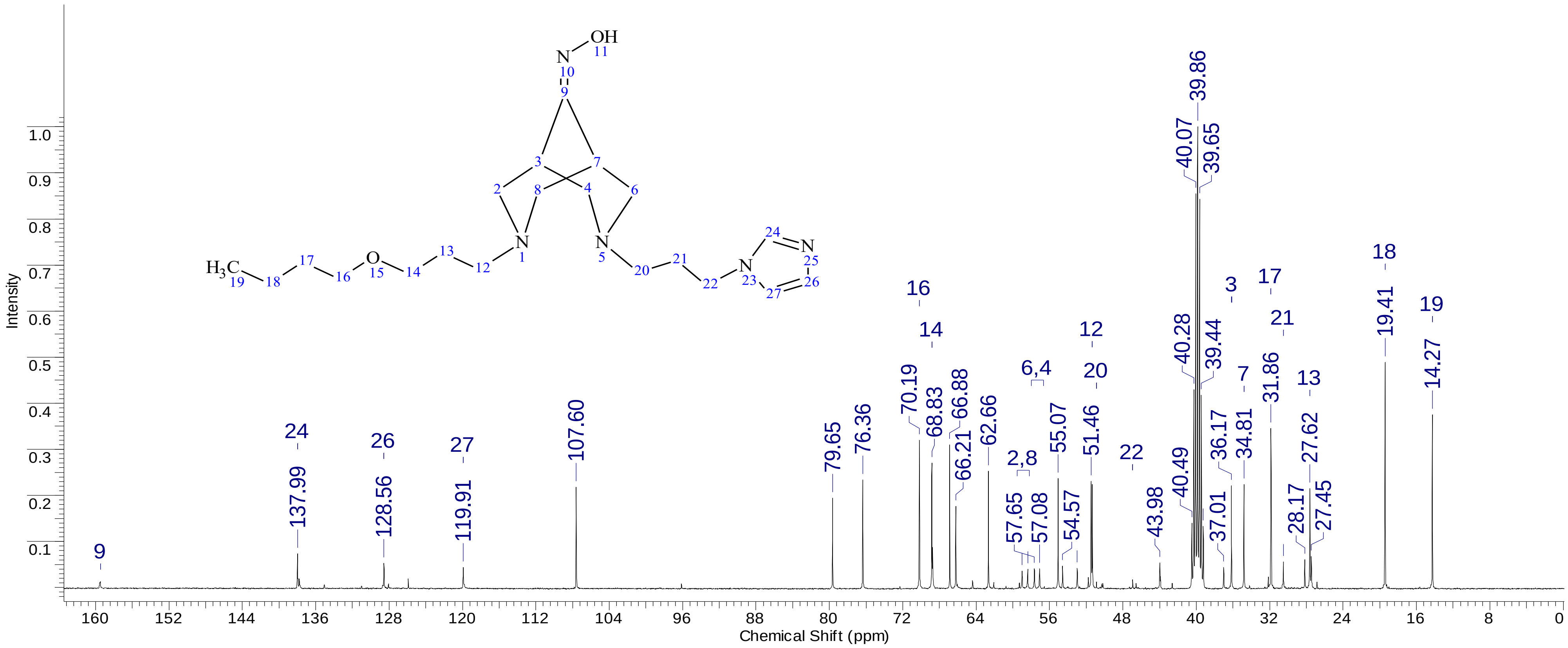

No.	Annotation	(ppm)	(Hz)	Height	No.	Annotation	(ppm)	(Hz)	Height
1	19	14.3	1434.0	0.3751	10	12	51.5	5173.0	0.2306
2	18	19.4	1951.6	0.4893	11	4, 6	57.1	5738.5	0.0412
3	13	27.6	2776.8	0.2145	12	2, 8	58.4	5868.8	0.0403
4	21	30.5	3066.3	0.0552	13	14	68.8	6919.3	0.2706
5	17	31.9	3202.4	0.3456	14	16	70.2	7055.4	0.3200
6	7	34.8	3499.5	0.2235	15	27	119.9	12053.8	0.0438
7	3	36.1-36.2	3635.6	0.2205	16	26	128.6	12923.1	0.0526
8	22	46.9	4421.5	0.0540	17	24	138.0	13804.5	0.0451
9	20	50.9	5160.5	0.2241	18	9	159.5	15052.8	0.0584

Figure S16. ^{13}C NMR spectrum of oxime of 3-(3-butoxypropyl)-7-[3-(1*H*-imidazol-1-yl)-propyl]-3,7-diazabicyclo[3.3.1]nonane-9-one (12) in DMSO-d$_6$

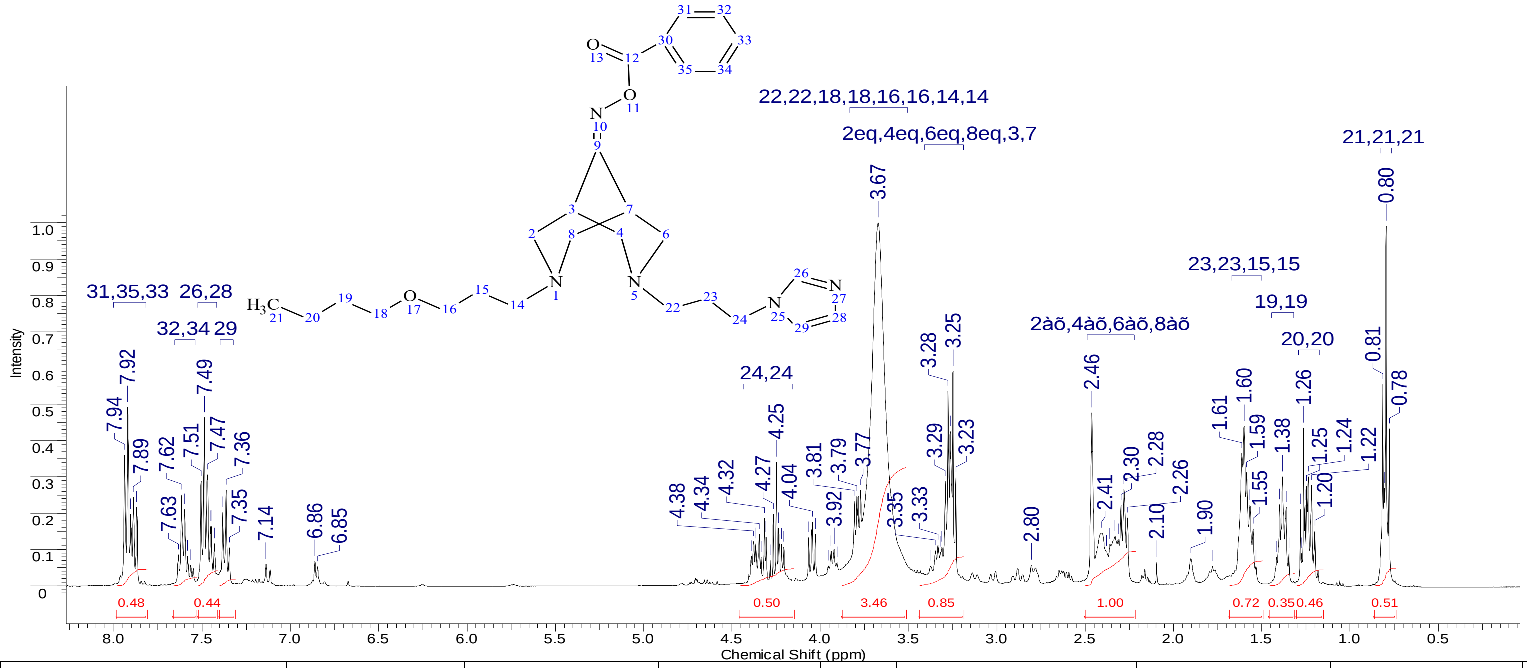

No.	Annotation	(ppm)	(Hz)	Height	No.	Annotation	(ppm)	(Hz)	Height
1	21	0.78-0.81	322.4	0.2691	7	14,16,18,22	3.67-3.81	1522.2	0.2341
2	20	1.20-1.26	501.9	0.2210	8	24	4.25-4.38	1712.7	0.0698
3	19	1.38	552.7	0.3004	9	29	7.35-7.36	2944.1	0.2644
4	23, 15	1.55-1.61	644.8	0.3651	10	26,28	7.47-7.51	3000.9	0.2882
5	2ax,4ax,6ax,8ax	2.26-2.46	984.1	0.4763	11	32,34	7.49-7.62	2993.1	0.4632
6	2eq,4eq,6eq,8eq,3,7	3.23-3.29	1316.1	0.2880	12	31,33,35	7.89-7.94	3173.5	0.3606

Figure S17. ^{1}H NMR spectrum of *O*-benzoyloxime of 3-(3-butoxypropyl)-7-[3-(1*H*-imidazol-1-yl)propyl]-3,7-diazabicyclo[3.3.1]-nonane-9-one (13) in DMSO-d$_6$

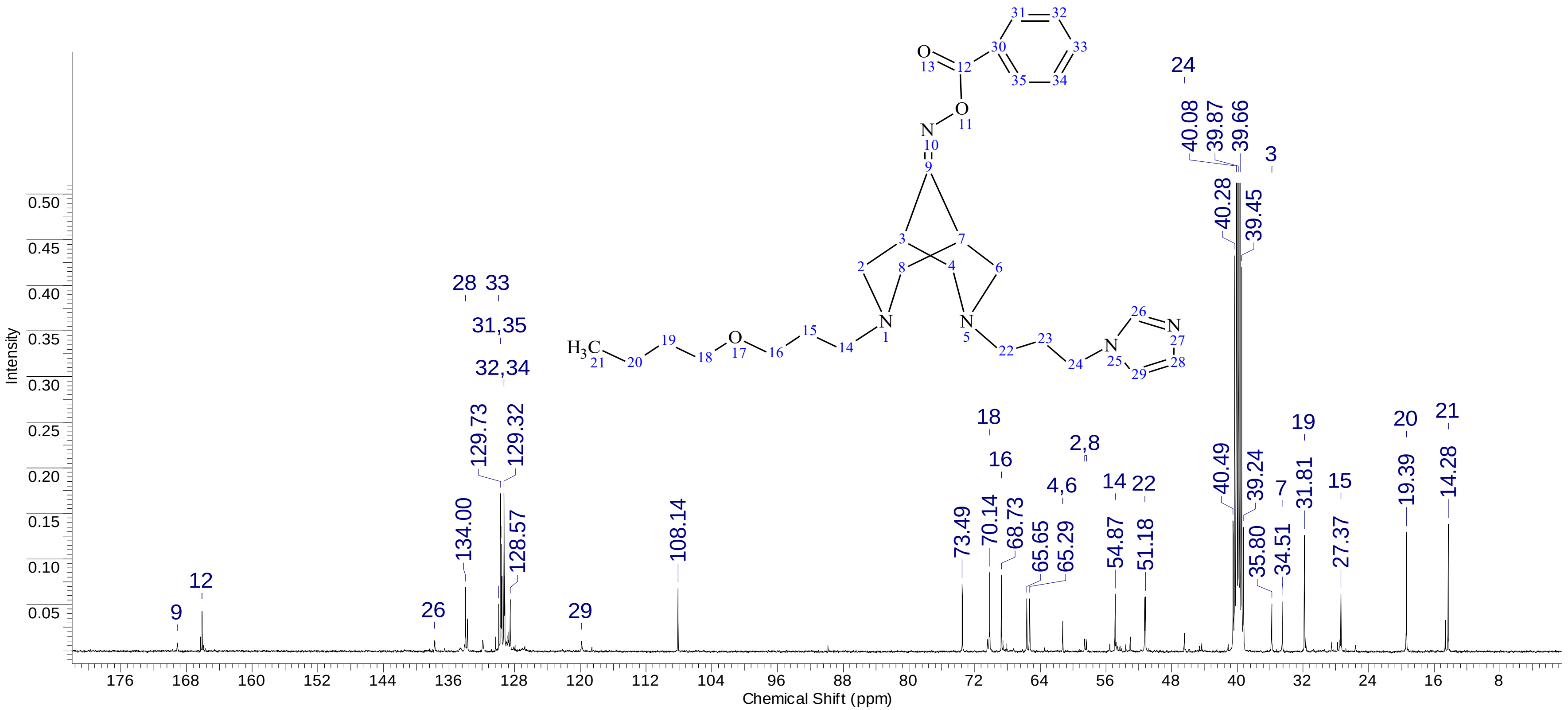

No.	Annotation	(ppm)	(Hz)	Height	No.	Annotation	(ppm)	(Hz)	Height
1	21	14.3	1435.9	0.1384	12	16	68.7	6908.8	0.0822
2	20	19.4	1947.7	0.1294	13	18	70.1	7050.6	0.0853
3	15	27.4	2750.9	0.0615	14	29	119.9	12054.2	0.0453
4	19, 23	31.8	3197.6	0.1259	15	32, 34	129.3	12999.8	0.1723
5	7	34.5	3468.8	0.0535	16	31, 35	129.7	13041.0	0.1715
6	3	35.8	3599.2	0.0506	17	33	130.0	13104.8	0.5893
7	24	46.4	4650.4	0.0452	18	28	134.0	13470.4	0.0685
8	22	51.3	5153.8	0.0580	19	26	137.8	13804.5	0.0451
9	14	54.9	5516.1	0.0610	20	12	166.1	16705.9	0.0584
10	2, 8	58.3-58.6	5878.6	0.0147	21	9	169.1	16985.2	0.0478
11	4, 6	61.3	6148.8	0.1475					

Figure S18. ^{13}C NMR spectrum of *O*-benzoyloxime of 3-(3-butoxypropyl)-7-[3-(1*H*-imidazol-1-yl)propyl]-3,7-diazabicyclo[3.3.1]-nonane-9-one (13) in DMSO-d$_6$
